# Prevalence of Metabolic Syndrome in Liver Transplant Recipients in Iran

**Published:** 2016-05-01

**Authors:** S. J. Masoumi, Z. Mazloom, A. Rezaianzadeh, S. Nikeghbalian, S. A. Malek-Hosseini, H. Salahi

**Affiliations:** 1 *School of Nutrition and Food Sciences, Shiraz University of Medical Sciences, Shiraz, Iran*; 2 *Nutrition and Food Sciences Research Center, Shiraz University of Medical Sciences, Shiraz, Iran*; 3 *Gastroenterohepatology Research Center, Shiraz University of Medical Sciences, Shiraz, Iran*; 4 *Department of Epidemiology, Shiraz University of Medical Sciences, Shiraz, Iran*; 5 *Department of Surgery, Shiraz University of Medical Sciences, Shiraz, Iran*; 6 *Transplant Research Center, Shiraz University of Medical Sciences, Shiraz, Iran*

**Keywords:** Metabolic syndrome, Transplant recipients, Hyperglycemia, Hyperlipidemias, Liver transplantation, Prevalence

## Abstract

**Background:**

Metabolic syndrome (MetSx) is common among liver transplant recipients. It contributes to morbidity and mortality.

**Objective:**

To determine the prevalence of MetSx in patients undergoing liver transplantation (LTx) in Iran.

**Methods:**

202 liver transplant recipients of both sexes completed this study. Relevant information including age, sex, the underlying disease, systolic and diastolic blood pressure, waist circumference, fasting serum levels of blood sugar (FBS), triglyceride (TG), and HDL-cholesterol were measured. The prevalence of MetSx was evaluated at 1, 3, 6, 9, and 12 months after LTx.

**Results:**

The prevalence of MetSx was 36.6% after 1 month that decreased to 28.2% after 12 months of follow-up. The lowest prevalence of MetSx (27.7%) was observed 9 months after LTx. Our data showed a decrease in TG and an increase in HDL-C level and no significant changes in blood pressure, waist circumference and FBS during the study period.

**Conclusion:**

The prevalence of MetSx after LTx is high when compared to the normal population. It seems that a change in diet after transplantation may affect the prevalence of MetSx.

## INTRODUCTION

Metabolic syndrome (MetSx) is a compilation of metabolic manifestations of obesity defined by a combination of increased abdominal girth, hypertension, hyperglycemia, and dyslipidemia [1, 2]. This disorder represents a number of cardiometabolic risk determinants including central obesity, insulin resistance, hypertension, and dyslipidemia [3, 4]. MetSx is one of the most common complications after liver transplantation (LTx). Obesity and MetSx are affecting almost every aspect of LTx including the type and incidence of post-LTx complications [5]. Several studies showed that MetSx existed in 40%–58% of patients followed for six months after LTx [6, 7]. 

Primary defects in energy balance that cause obesity (visceral adiposity in particular) are sufficient to drive all aspects of the syndrome. Increased free fatty acids and accumulation of fat in certain organs are mediators of obesity and insulin resistance. Obesity also leads to proinflammatory and prothrombotic states that potentiate atherosclerosis. Pathways leading directly from adiposity to the genesis of dyslipidemia and hypertension have been elucidated and reported [8]. Recent knowledge implies a role for fat-derived “adipokines” including TNF and adiponectin, as pathogenic contributors or protective factors [8].

The incidence of new-onset diabetes mellitus (NOD) after orthotopic LTx in adult transplant recipients reported to be 25.8% [9], while MetSx reported to be 51.9% after LTx [7]. In another study, it was shown that the absolute prevalence of diabetes mellitus (DM) rose from about 15% prior to LTx to 30%–40% after LTx [5]. Kuo and his colleagues reported that the NOD after LTx occurred in 26% of patients without documented diabetes before transplantation in the United Network for Organ Sharing database [10]. Considering the other components of MetSx, dyslipidemia has been detected in approximately 50%–70% of patients after LTx [11]. Hypertension also increased from 15% in pre-transplantation patients to 60%–70% in those post-transplantation [5]. Obesity and increased waist circumference were also seen commonly after LTx [12, 13]. The greatest weight gains occurred within the first six months after transplantation [5]. Needless to say, the impact of MetSx in LTx recipients is substantial, and it may cause graft rejection after transplantation. The present study was undertaken to determine the prevalence of MetSx in patients undergoing LTx in Iran. 

## PATIENTS AND METHODS

Patients

Two-hundreds and two adult LTx recipients from Shiraz Organ Transplantation Center in Nemazee Hospital affiliated to Shiraz University of Medical Sciences, Shiraz, Iran, who had no previous history of acute cardiovascular diseases (CVD) or cancer before LTx, were enrolled in this study. Patients who had recurrence of cirrhosis or chronic ascites and pregnant women were excluded from the study. The study was approved by the Ethics Committee of Shiraz University of Medical Sciences, Shiraz, Iran. A written informed consent was obtained from each patient. All clinical evaluations were performed at Organ Transplantation Center; all tests were done at Nemazee Hospital.

Follow up

Patients were followed for 12 months. The National Heart, Lung, and Blood Institute/American Heart Association (NHLBI/AHA) clinical identification of MetSx was considered for the diagnosis of MetSx [14]. The diagnosis depended on observation of at least three of the following criteria: (i) waist circumference in women >88 cm and in men >102 cm; (ii) fasting blood sugar (FBS) >100 mg/dL; (iii) blood pressure (BP): systolic >130 mm Hg, diastolic >85 mm Hg; (iv) high-density lipoprotein cholesterol (HDL-C) in women <50 mg/dL and in men <40 mg/dL; and (v) triglyceride (TG) level >150 mg/dL. The demographic information and medical history were also recorded for each patient. 

Clinical Evaluations

All clinical evaluations were performed at Organ Transplantation Center of Nemazee Hospital. The follow up was monthly for the first six months, and then at 9^th^ and 12^th^ months. Waist circumference was measured at the umbilicus level with the patient standing as an index of central fat accumulation. BP was also determined for each patient.

Biochemical Analyses

Blood samples were drawn from each subject after an overnight fasting. Serum samples were stored at 80 °C until analyses. Fasting plasma glucose was measured on the day of blood collection by the enzymatic colorimetric method using glucose oxidase. Serum TG concentration was measured by commercially available enzymatic reagents adapted to an autoanalyzer. HDL-C was measured after precipitation of the apolipoprotein B-containing lipoproteins with phosphotungistic acid. 

Statistical Analysis

Statistical analyses were performed by SPSS^®^ for Windows^®^ ver 20.0 (SPSS Inc., Chicago, IL, USA). The results are presented as mean±SD.

## RESULTS

**Figure 1:**
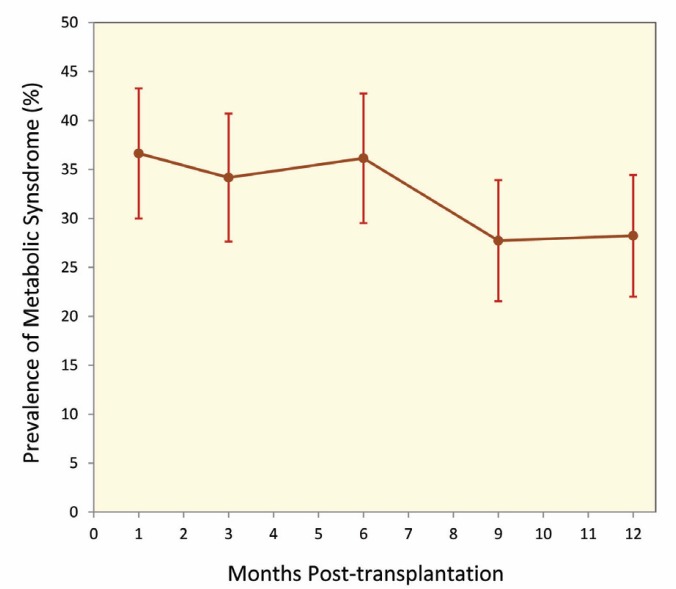
Prevalence of metabolic syndrom during study period

The baseline characteristics data and underlying diseases/disorders of the LTx recipients are presented in Table 1. The male to female ratio in our study was nearly 2:1 (126 male and 76 female patients). The most frequent underlying diseases in LTx patients were DM and hyperlipidemia (six and three patients, respectively), where hypertension and renal diseases were the least (two and one patient, respectively). Analyses of the biochemical variables, including FBS, TG, and HDL-C level are presented in Table 2. TG concentration decreased from 188.6±77.2 mg/dL on the 1^st^ month to 136.5±67.2 mg/dL at the 12^th ^month. HDL-C also increased considerably from 40.6±12.0 mg/dL at the 1^st^ month to 43.6±12.9 mg/dL at the 12^th^ month. Waist circumference and systolic and diastolic blood pressure did not change significantly during the 12^th^ month of the study (Table 3). The highest prevalence of MetSx (36.6%: 95% CI: 30.0%–43.3%) was recorded at the 1^st^ month after transplantation where the lowest prevalence (27.7%; 95% CI: 21.5%–33.9%) was at the 9^th^ month (Fig 1).

**Table 1:** Baseline characteristics and underlying disease/disorder of liver transplant recipients (n=202

Categories		n (%)
Sex	Female	76 (37.6)
	Male	126 (62.4)
DM	No	196 (97.0)
	Yes	6 (3.0)
HT	No	200 (99.0)
	Yes	2 (1.0)
HL	No	199 (98.5)
	Yes	3 (1.5)
CVD	No	202 (100)
	Yes	0 (0)
RD	No	201 (99.5)
	Yes	1 (0.5)
Cancer	No	202 (100)
	Yes	0 (0)

* DM: Diabetes mellitus; HT: Hypertension; HL: Hyperlipidemia; CVD: Cardiovascular diseases; RD: Renal diseases

**Table 2:** Biochemical analysis of patients after liver transplantation (n=202

Variables	Follow up month				
	1^st^	3^rd^	6^th^	9^th^	12^th^
FBS (mg/dL)	110.2±53.7	96.4±28.5	100.2±36.6	100.5±40.46	102.5±42.4
TG (mg/dL)	188.6±77.2	167.5±76.9	155.2±75.0	142.0±63.9	136.5±67.2
HDL-C (mg/dL)	40.6±12.0	44.4±11.9	44.8±12.1	43.6±11.7	43.6±12.9

FBS: Fasting blood sugar; TG: Triglyceride; HDL-C: High-density lipoprotein cholesterol

**Table 3:** Analysis of clinical variables after liver transplantation (n=202

Variables	Follow up month				
	1^st^	3^rd^	6^th^	9^th^	12^th^
Blood pressure (mm Hg)					
Systolic	118.4±11.9	120.5±11.1	121.1±11.1	119.5±10.6	119.2±10.2
Diastolic	76.6±7.9	77.3±7.2	78.5±7.0	78.2±6.8	77.3±7.4
Waist circumference (cm)	86.1±9.8	86.1±9.7	86.6±9.2	85.9±8.8	88.1±10.5

## DISCUSSION

MetSx is common among LTx recipients. Although the cause of post-LTx MetSx is not clear, post-transplantation weight gain together with the side effects of immunosuppressive drugs could play a role in induction of MetSx. This study assessed the prevalence of MetSx after LTx in patients hospitalized in the Organ Transplantation Center, Shiraz, Iran. Although, a slight tendency to decline in the prevalence of MetSx was seen in LTx patients during the study period, this decline was not sharp. A reduction in TG and an increase in HDL-C level were also seen in the transplant recipients. However, no changes were noticed in the BP, waist circumference and FBS during the study period.

Considering the five metabolic components of MetSx—obesity, hyperglycemia, hypertriglyceridemia, hypertension, and low HDL-C level—we found obesity in one-third to one-half of patients with normal weight at the time of LTx [13]. Hanouneh, *et al*, in their study evaluated the underlying mechanisms of this phenomenon and demonstrate that steroids and immunosuppressive drugs, which are commonly used after LTx, are responsible for this increase [12]. As much as the obesity affects the incidence of DM, hyperlipidemia, and hypertension (other components of MetSx), there has been a lot of focus on NOD after LTx in recent years. 

In a study performed by Kuo, *et al*, the NOD-related risk factors were evaluated. It was found that old age (more than 60 years), body mass index (BMI) more than 25 kg/m^2^, HCV infection, cirrhosis, and use of methylprednisolone, tacrolimus (TAC), and steroids in recipients and a history of diabetes in donors are independent risk factors for the development of NOD after LTx [10]. Liver vagotomy, which occurred during LTx, and immunosuppressive drugs can induce insulin resistance and development of NOD [5]. 

The prevalence of MetSx among Iranian population is about 30% [15]. Clearly, that prevalence of MetSx can be changed in response to diseases affecting liver [16]. Hanouneh, *et al*, reported a 50% rate of MetSx in their patients after LTx [17]. This value was reported as 58% by Laryea, *et al* [6]. Francioso, *et al*, and Sprinzl, *et al*, reported the prevalence of post-LTx MetSx to be 43% and 45%, respectively [18, 19]; the prevalence in our study was 36.1% six months post-LTx. Comparing the findings of this study with the Canadian and Italian studies, revealed that the prevalence of MetSx is lower in Iran. This may be attributed to variations in the studied population, life style and the selected criteria. It seems that the Iranian proposed criteria adopted by Iranian National Committee of Obesity would be better diagnostic criteria for evaluation of MetSx among Iranian population [20]. In another study, it was shown that the clinical and biochemical features of MetSx were independent from etiology of pre-LTx liver disease [21]. However, in a recent study by De Luca and his colleagues, it was reported that patients transplanted for etiologies other than non-alcoholic steatohepatitis, typically gained weight and often developed DM, hypertension and dyslipidemia as a consequence of immunosuppressive therapy and a resultant MetSx [22]. 

Sprinzl and his colleagues reported the new-onset and total prevalence of MetSx after LTx that were 32.9% and 45.3%, respectively in their study [23]. MetSx was also reported in 51.9% of patients after LTx by Laish and his colleagues in 2011 [7]. Although the prevalence of MetSx in these studies were higher than what we detected in the present study (36.6%), this could be due to differences in the underlying diseases and the variation in patients’ life style, needless to say that in spite of the differences, the prevalence of MetSx in our patients was in line with many other studies [6, 18, 23] that demonstrated that approximately one-third of the patients suffered from MetSx after LTx. An interesting finding of this study was the decline in the prevalence of MetSx after 12 months of LTx; other studies did not report any decrease during their follow up [24]. These differences may be due to different definitive criteria for evaluation of MetSx and its components. 

DM, hypertriglyceridemia, hypertension, and low levels of HDL-C were noticed in patients with MetSx in the present study. Similar findings were also reported in previous studies for DM [25-31], dyslipidemia [29, 30, 32-34], abdominal obesity [29, 30, 34], and hypertension [29, 30, 33, 35] as well. 

A clear decrease in TG and an obvious increase in HDL-C level were observed during the 12 months of follow up. Liver is the main organ where lipid synthesis and degradation are occurred. Also, it involves fat excretion by bile acid synthesis. Any changes in its normal function can thus disturb the lipid balance and alter the lipid profile. Our findings could be attributed to an enhanced lipid turnover and body fluid re-compensation during hepatic recovery, but it might also be explained by a post-interventional catabolic phase as well [23]. 

In conclusion, the prevalence of MetSx after LTx is still higher compared to the normal population previously reported in Iran and other countries. It seems that a change in diet and physical activity of LTx patients may affect the prevalence of MetSx. There is a need for further interventional research on these patients to decrease the prevalence of MetSx.
